# Effective adjuvantation of nanograms of influenza vaccine and induction of cross-protective immunity by physical radiofrequency adjuvant

**DOI:** 10.1038/s41598-022-25605-4

**Published:** 2022-12-08

**Authors:** Zhuofan Li, Xinliang Kang, Ki-Hye Kim, Yiwen Zhao, Yibo Li, Sang-Moo Kang, Xinyuan Chen

**Affiliations:** 1 Biomedical & Pharmaceutical Sciences, College of Pharmacy, University of Rhode Island, 7 Greenhouse Road, Avedisian Hall, Room 480, Kingston, RI 02881 USA; 2 Center for Inflammation, Immunity & Infection, Institute for Biomedical Sciences, Georgia State University, Atlanta, GA 30302 USA

**Keywords:** Vaccines, Adjuvants

## Abstract

Novel adjuvants are highly demanded to aid in development of improved or new vaccines against existing or emerging infectious diseases. Considering commonly used Alum and MF59 adjuvants induce tissue stress and release of endogenous danger signals to mediate their adjuvant effects, physical modalities may be used to induce tissue stress and endogenous danger signal release to enhance vaccine-induced immune responses. Furthermore, physical adjuvants are less likely to induce significant systemic adverse reactions due to their localized effects. Recently we found non-invasive radiofrequency (RF) pretreatment of the skin could significantly enhance intradermal vaccine-induced immune responses in murine models that included pandemic influenza vaccine, pre-pandemic vaccine, and influenza internal antigen vaccine. It remained to be explored whether the physical RF adjuvant (RFA) could be used to boost seasonal influenza vaccination, spare vaccine doses, and induce cross-protective immunity. This study found the physical RFA could significantly enhance seasonal influenza vaccine-induced immune responses against each viral strain and robustly enhance low-dose (nanograms) H3N2 vaccine-induced immune responses and protection in murine models. RFA also induced cross-protective immunity against heterologous and heterosubtypic influenza viruses. Further studies found heat shock protein 70 (inducible endogenous danger signal) and myeloid differentiation primary response 88 adaptor played a crucial role in dose-sparing effects of RFA. These data strongly support further development of the physical RFA to boost influenza vaccination.

## Introduction

Adjuvants are traditionally defined as substances added to vaccines to enhance vaccine-induced immune responses. Adjuvants have multifaceted roles in vaccine development, for example, enabling the development of effective vaccines against infectious diseases and cancer, inducing protective immunity in the elderly, young children, and immunocompromised populations, sparing vaccine doses to immunize more people at risk (e.g., first-responders during a pandemic), or inducing cross-protective immunity against mutated viral strains^1^. Considering vaccines are mainly given to healthy populations, adjuvants need to have good safety profiles before they can be approved for human use. For this reason, many powerful adjuvants, such as complete and incomplete Freund’s adjuvant, lipopolysaccharide (LPS), a variety of cytokines, are not approved for human use due to their high risk to induce local or systemic adverse reactions^2^.

Aluminum salt-based adjuvant (Alum) was first used in 1930s and has been the most widely used adjuvant in human vaccines^3^. Several new adjuvants were approved in the last 2–3 decades that included squalene emulsion adjuvants (MF59, AS03), Adjuvant System adjuvants (AS01, AS04), and CpG oligonucleotide (CpG) 1018 adjuvant^4^. Accompanied with this advance, the underlying mechanisms of vaccine adjuvants were also gradually uncovered, thanks to the increasing understanding on how the adaptive immune systems are activated in the absence of pathogen infections^5,6^. It was found endogenous danger signals released under specific tissue stress could activate innate immune systems to support the adaptive immunity^5,6^. Previously it was believed mainly foreign materials, such as pathogen-associated molecular patterns (PAMPs), could activate innate immune systems to support the adaptive immunity^5,6^.

Endogenous danger signals are diverse types of small chemicals and macromolecules that exist in physiological conditions and can release under tissue stress to activate innate immune systems^7^. Common endogenous danger signals include uric acid, ATP, double-strand DNA (dsDNA), and heat shock proteins (HSPs)^7^. Uric acid, ATP, and dsDNA exist in physiological conditions in the cytosol, mitochondria, or cell nucleus^7^. HSPs exist in diverse cellular compartments (e.g., cytosol, endoplasmic reticulum, mitochondria) or induced by a variety of stimuli not limited to heat shock^7,8^. Extracellular release of these molecules activates innate immune systems and sometimes is linked to pathological mechanisms of autoimmune diseases, such as arthritis and gout^9^. Several danger signals have been found to mediate adjuvant effects of Alum and MF59. Both adjuvants have no specific cellular receptors to mediate their adjuvant effects. Alum adjuvant was once thought to retain antigens at local injection sites to mediate its adjuvant effects. Later it was found the ‘antigen depot’ was not critical to alum adjuvant effects since removal of the injection site as early as 2 h had no appreciable effects on antigen-specific T and B cell responses^10^. Both adjuvants were found to induce tissue stress, such as cell deaths and apoptosis, at local injection site or in draining lymph nodes^11,12^. Kool et al. found Alum adjuvant could induce uric acid release from Alum-injected tissues and activate NLRP3 inflammasome to mediate local inflammatory responses^13^. ATP was found to release extracellularly from MF59-injected tissues and play a crucial role in its adjuvant effects^14^. These studies indicated induction of tissue stress and release of endogenous danger signals might enhance vaccine-induced immune responses.

Radiofrequency (RF) is highly alternating electromagnetic waves and can be used to heat tissue by induction of oscillation of water molecules within the tissue. RF has been broadly used in skin resurfacing by generation of thermal heating to cause collagen denaturation and stimulate neo-collagen synthesis^15^. RF has been also used to ablate tumor tissues in tumor therapy or nerve fibers in pain management^16,17^. Recently, we explored non-invasive RF treatment of the skin to induce thermal stress to enhance intradermal (ID) vaccine-induced immune responses. We found the physical RF adjuvant (RFA) showed potent adjuvant effects to boost influenza pandemic 2009 H1N1, split-virion H5N1, and recombinant nucleoprotein (NP) and matrix protein 1 (M1) vaccination in murine models^18–20^. RFA was further found to elicit transient low-level local inflammation, while chemical adjuvants (Alum, AddaVax, monophosphoryl lipid A (MPL)) induced intense and persistent local inflammation^20^. RFA was also less likely to induce systemic or long-term side effects due to its well-controlled local effects and the physical nature of the adjuvant. Despite these promising results, it remained to be explored whether RFA could enhance seasonal influenza vaccination, spare vaccine doses, and induce cross-protective immunity. This study explored the various adjuvant effects of RFA on influenza vaccination in murine models.

## Materials and methods

### Reagents

Monovalent 2009 H1N1 influenza (pdm09) vaccine (NR-20083), trivalent inactivated influenza vaccine (TIV, 2011–2012) (NR-36747), and influenza A/Puerto Rico/8/1934 (H1N1) viruses (NR-28652, abbreviated as PR8) were obtained from BEI Resources (Manassas, VA). Influenza A/California/07/2009 (H1N1) viruses (FR-201), A/Victoria/210/2009 X-187 (H3N2) viruses (FR-644), influenza B/Brisbane/60/2008 viruses (Victoria Lineage) (FR-177), and recombinant hemagglutinin antigen (rHA) of influenza A/California/07/2009 (H1N1) (FR-559) were obtained from International Reagent Resource (IRR, Manassas, VA). AddaVax adjuvant was purchased from Invivogen (vac-adx-10, San Diego, CA). Chicken red blood cells (RBCs) (10100768) were purchased from Charles River Laboratories (Wilmington, MA).

### Mice

C57BL/6 and BALB/c mice (6–8 weeks old, male) were purchased from Charles River Laboratories (Wilmington, MA). MyD88 knockout (KO) mice (009088) were obtained from Jackson Laboratory (Bar Harbor, ME). HSP70 KO mice (cryo-preserved, 030411-MU) were ordered from Mutant Mouse Resource & Research Centers (MMRRC) at University of Missouri. One litter of heterologous HSP70 mice were received and self-bred to obtain HSP70 WT and HSP70 KO mice for self-breeding to obtain sufficient mice for use in this study. Animals were housed in animal facilities of the University of Rhode Island (URI) and anesthetized by intraperitoneal injection of Ketamine (80 mg/kg) and Xylazine (10 mg/kg) for hair removal, RF treatment, and immunization. Animal experiments involving influenza viruses were conducted in animal biosafety level 2 (ABSL2) facility of URI. All animal procedures were approved by the Institutional Animal Care and Use Committee of URI and conducted in accordance with National and Institutional Guidelines and Regulations. Animal experiments were reported in accordance with the ARRIVE guidelines.

### RF device

A cosmetic fractional bipolar RF device of ~ 1 MHz (Norlanya Technology Co., Hong Kong, China) equipped with 12 × 12 array of microelectrodes in 2 × 2 cm^2^ area was used. This device has three energy settings (low, medium, high) and high-energy setting was used in this study to induce significant tissue stress in 1–2 min. For RF treatment, a thin layer of ultrasound coupling medium was applied on the skin surface as recommended by the manufacturer and RF device was then firmly pressed to allow treatment tips to have a close contact with skin surface.

### Immunization

Hair on the lateral back skin of mice was removed one day before experiment by clipping and application of hair-removal lotion. Next day, hair-removed skin was exposed to RF or sham treatment followed by ID injection of vaccine into RF- or sham-treated skin as in our previous reports^18–20^. In chemical adjuvant groups, mice were intramuscularly injected with the same amount of vaccine in the presence of AddaVax (50%, vol/vol) except otherwise specified.

### Inactivated H3N2 split vaccine

For preparation of a monovalent split-virion H3N2 vaccine, H3N2 viruses (A/Philippines/2/82) were expanded in 10-day-old embryonated hen’s eggs and then inactivated by treating with 1% neutral formalin. The inactivated H3N2 viruses were harvested by ultracentrifugation and then treated with 1% Triton X-100 to disrupt virus particles followed by dialysis against phosphate-buffered saline (PBS) as in our prior report^21^. Detergent-compatible protein assay kit (Bio-Rad) was used to determine protein concentration of the split-virion H3N2 vaccine. Hemagglutinin antigen (HA) content is estimated to be approximately 29–30% of total influenza virion proteins^22,23^.

### Influenza viral challenge

H3N2 viruses (A/Philippines/2/82) were expanded in 10-day-old embryonated hen’s eggs and the allantoic fluid was collected for use in challenge studies. Fifty percent lethal dose (LD50) in mice was determined following a well-established method^24^. Mouse-adapted pdm09 viruses and influenza A/Puerto Rico/8/34 H1N1 (PR8) viruses were prepared as in our prior reports^20,25,26^. Mice were intranasally challenged with different doses of H3N2, pdm09, or PR8 viruses under anesthesia. Mouse body weight and survival were monitored daily for 14 days. The mice were euthanized and considered dead if their body weight loss was more than 25%.

### HI titer

Serum hemagglutination inhibition (HI) titer was measured as in our previous report^25^. In brief, serum samples were incubated with receptor-destroying enzyme II, heat inactivated, and then incubated with chicken RBCs to remove non-specific binding. Serum samples were then subjected to two-fold serial dilutions and incubated with four hemagglutinating units of influenza viruses. Viruses were propagated in 9–11-day embryonic eggs for use in this study. Chicken RBCs were added and HI titer was determined as the reciprocal of the highest dilution that completely inhibited agglutination of chicken RBCs.

### ELISA antibody titer

Serum antibody titer was measured by enzyme-linked immunosorbent assay (ELISA) as in our previous report^25^. In brief, 96-well ELISA plates were coated with 2 µg/ml rHA at 4 °C overnight. After blocking with 5% non-fat milk, two-fold serial dilutions of immune sera were added and incubated at room temperature for 90 min. After washing in PBS supplemented with 0.05% Tween 20 (PBST), horseradish peroxidase (HRP)-conjugated sheep anti-mouse IgG secondary antibodies (1:2500, NA931, GE Healthcare Life Sciences) were added and incubated at room temperature for 1 h. After washing in PBST, TMB substrates were added and reactions were then stopped by addition of 3 N H_2_SO_4_. Optical absorbance (OD_450nm_) was determined using a microplate reader (Molecular Device). Serum antibody titer was defined as the reciprocal dilution factor that resulted in OD_450nm_ ~ 3 times higher than the background values.

### Statistics

Values were expressed as Mean ± SEM (standard error of the mean). Student’s t-test was used to analyze differences between groups and one-way ANOVA with Tukey’s multiple comparison test was used to compare differences for more than 2 groups except otherwise specified. Two-way ANOVA with Tukey’s multiple comparison test was used to compare body weight difference at different time points between groups except otherwise specified. Log-rank (Mantel-Cox) test with Bonferroni correction was used to compare differences of survival between groups. P value was calculated by PRISM software (GraphPad, San Diego, CA) and considered significant if it was less than 0.05.

## Results

### RFA boosts seasonal influenza vaccination

**Figure 1:**
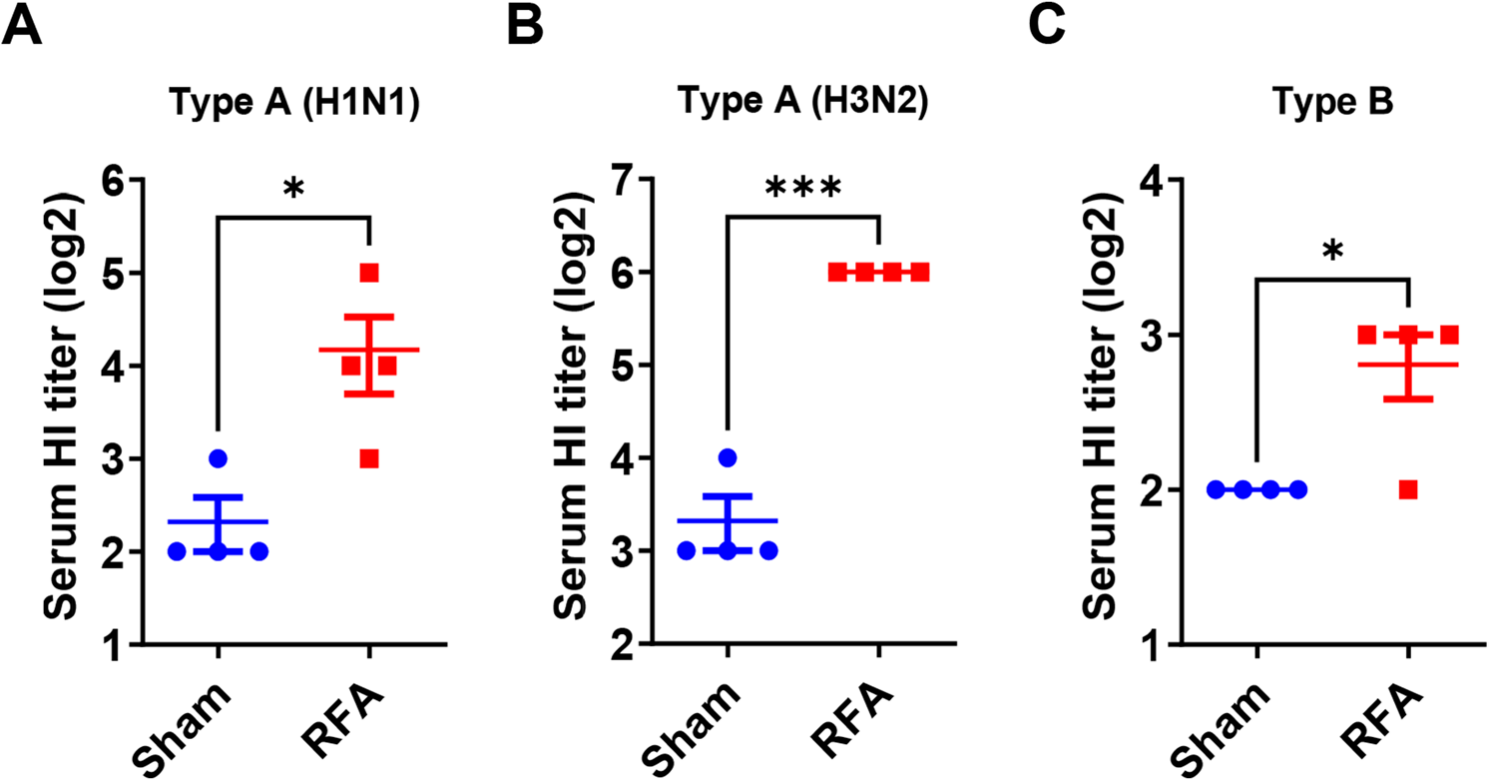
RFA enhances TIV-induced HI titer. C57BL/6 mice were subjected to RFA or sham treatment followed by ID injection of 20 µl TIV (equivalent to 0.3 µg HA/strain) into RF or sham-treated skin. Serum HI titer against each viral strain was measured three weeks later. One-tailed student’s t-test was used to compare differences between RFA and sham groups. n = 4. *p < 0.05; ***p < 0.001.

Our previous studies found the physical RFA could enhance the efficacy of influenza pandemic 2009 H1N1 vaccine, split virion H5N1 vaccine, and intracellular NP/M1 vaccine^18–20^. It remained to be explored whether RFA was also effective to boost seasonal influenza vaccination. Here, we used TIV (2011–2012) comprised of influenza A/California/07/2009 X-179A H1N1 (pdm09), A/Victoria/210/2009 X-187 H3N2 (an A/Perth/16/2009-like virus), and B/Brisbane/60/2008 as an example to explore RFA effects in murine models. Briefly, mice were subjected to RFA or sham treatment followed by ID injection of TIV into RF or sham-treated skin. Serum HI titer was measured three weeks later. As shown in Fig. 1, RFA significantly increased serum HI titer against all viral strains. RFA increased serum HI titer against influenza A H1N1, H3N2, and influenza B viruses by 3.6, 6.4, 1.75 folds, respectively (Fig. 1). This result indicated RFA could increase TIV-induced HI titer against each viral strain though to different extents.

### RFA enhances H3N2 vaccination

**Figure 2:**
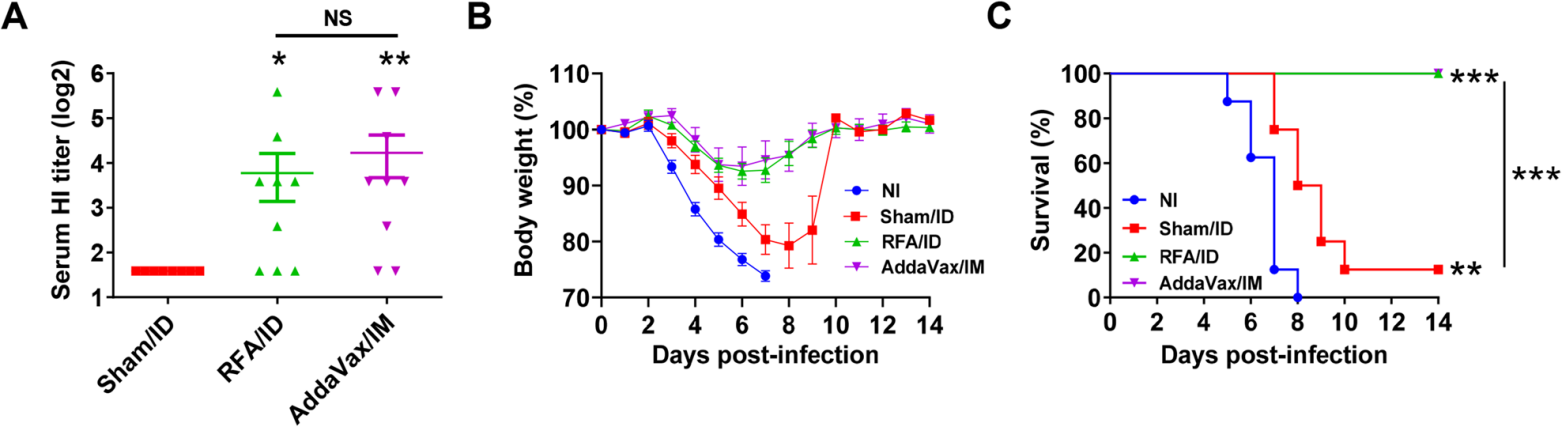
RFA boosts monovalent H3N2 vaccination. BALB/c mice were subjected to RF or sham treatment followed by ID delivery of 0.1 µg H3N2 vaccine (HA equivalent) into RF or sham-treated skin or intramuscularly injected with the same vaccine dose in the presence of AddaVax or left non-immunized (NI). Serum HI titer was measured 3 weeks later and shown in (**A**). Mice were then challenged with a lethal dose of homologous H3N2 viruses (50 × LD50). Body weight change (**B**) and survival (**C**) were monitored daily for 14 days. n = 8–9. One-way ANOVA with Dunn’s multiple comparison test was used to compare differences among groups in (**A**). Log-rank test with Bonferroni correction was used to compare differences of survival between NI and other groups or otherwise specified in (**C**). *p < 0.05; **p < 0.01; ***p < 0.001. *NS* not significant.

Seasonal influenza vaccines were found to have a lower effectiveness against H3N2 strain post the 2009 H1N1 influenza pandemic^27^. H3N2 strain also has a long-term antigenic mutation rate 17 times higher than the pdm09 H1N1 strain and 5–6 times higher than the type B strain^27^. Thus, adjuvants effective to boost H3N2 vaccination are highly desired. The above studies found RFA was highly effective to enhance TIV-induced HI titer against H3N2 strain. Next, a monovalent split-virion H3N2 vaccine (A/Philippines/2/82) was used to confirm the above finding and also explore the protection against viral challenges. In addition, MF59-mimetic AddaVax adjuvant was included for comparison. In brief, mice were subjected to RF or sham treatment followed by ID delivery of H3N2 vaccine into RF or sham-treated skin or intramuscularly immunized with the same vaccine dose in the presence of AddaVax adjuvant or left non-immunized. AddaVax adjuvant was delivered into the muscle due to its high risk to induce significant skin reactions following ID delivery. As shown in Fig. 2A, ID immunization in the presence of RFA induced a similar HI titer to that induced by intramuscular (IM) immunization in the presence of AddaVax adjuvant. Serum HI titers in RFA/ID and AddaVax/IM groups were significantly higher than that in sham/ID group (Fig. 2A). Following lethal viral challenges, mice in NI and sham/ID groups lost more than 20% body weight and mice in RFA/ID and AddaVax/IM groups lost less than 10% body weight (Fig. 2B). Mice in RFA/ID and AddaVax/IM groups recovered to their original body weights on day 10 after challenge (Fig. 2B). All mice died in NI group and 7 out of 8 mice died in sham/ID group, while all mice survived in RFA/ID and AddaVax/IM groups (Fig. 2C). This study confirmed the high potency of RFA to boost H3N2 vaccination.

### Significant dose-sparing effects of RFA on H3N2 vaccination

**Figure 3:**
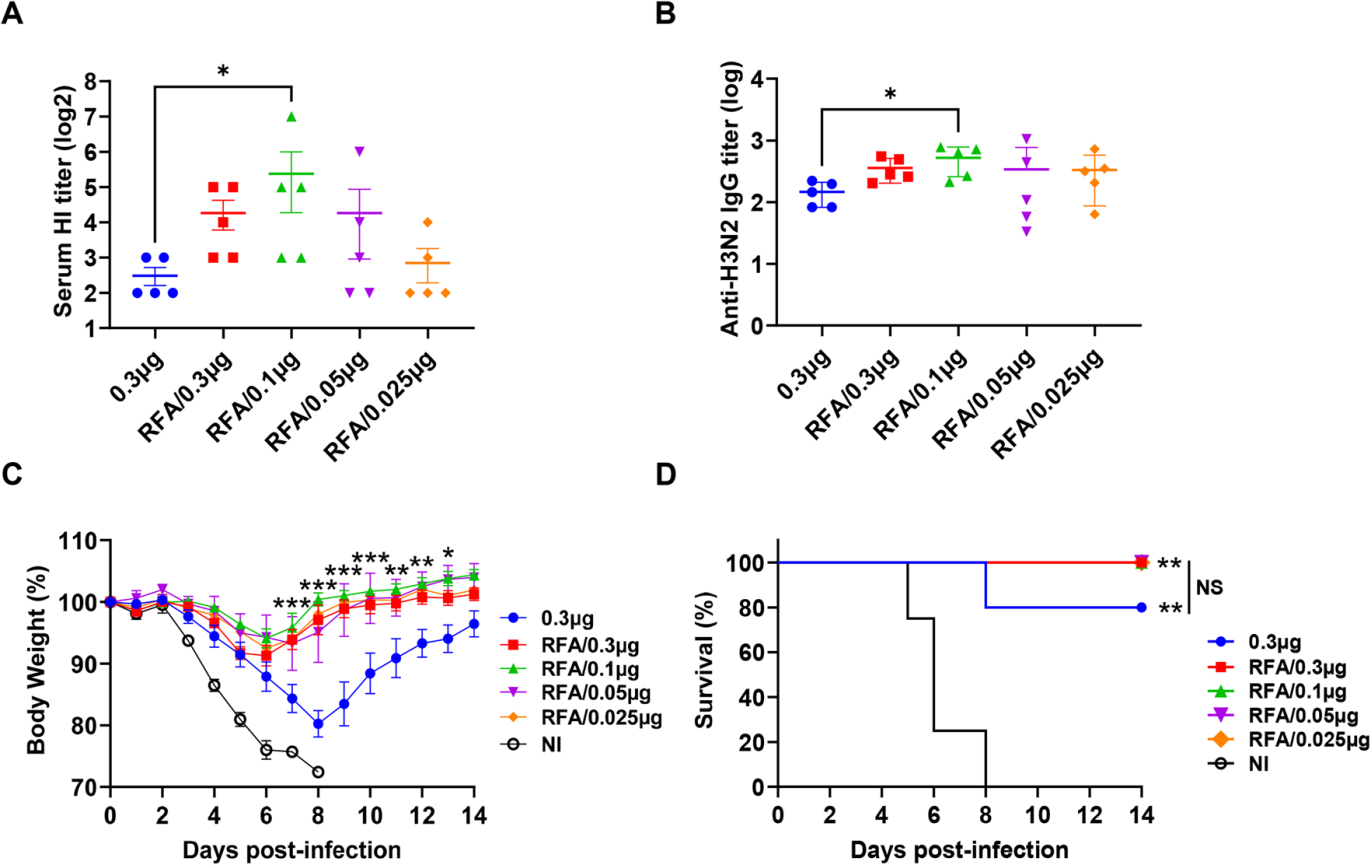
Dose-sparing effects of RFA in vaccine dose range (0.3–0.025 µg). BALB/c mice were intradermally injected with 0.3 µg H3N2 vaccine or subjected to RF treatment followed by ID injection of H3N2 vaccine of reducing doses (0.3–0.025 µg). Serum HI (**A**) and IgG titers (**B**) were measured three weeks later. Mice were challenged with 50 × LD50 of H3N2 viruses four weeks after immunization. Body weight change (**C**) and survival (**D**) were monitored daily for 14 days. n = 5. One-way ANOVA with Dunn’s multiple comparison test was used to compare differences among groups in (**A**) and (**B**). Two-way ANOVA with Dunnett’s multiple comparison test was used to compared differences between vaccine alone and RFA groups in (**C**). Log-rank test with Bonferroni correction was used to compare differences of survival between NI and other groups or otherwise specified in (**D**). *p < 0.05; **p < 0.01; ***p < 0.001. *NS* not significant.

Our previous study found reduction of pdm09 vaccine dose from 0.3 to 0.06 µg in the presence of RFA did not significantly reduce vaccine efficacy^20^, hinting potent dose-sparing effects of RFA on pdm09 vaccination. Here, we explored dose-sparing effects of RFA on H3N2 vaccination. In the first experiment, mice were subjected to ID delivery of 0.3 µg H3N2 vaccine or reducing H3N2 vaccine doses till 0.025 µg in the presence of RFA. As shown in Fig. 3A, H3N2 vaccine at all tested doses in the presence of RFA induced at least the same HI titer as H3N2 vaccine alone at 0.3 µg dose. Interestingly, H3N2 vaccine at 0.1 µg dose in the presence of RFA elicited significantly higher serum HI titer than H3N2 vaccine alone at 0.3 µg dose. Serum anti-H3N2 vaccine IgG titer showed the same trend. H3N2 vaccine of all doses in the presence of RFA elicited at least the same levels of anti-H3N2 IgG titer as compared to H3N2 vaccine alone at 0.3 µg dose (Fig. 3B). H3N2 vaccine at 0.1 µg dose in the presence of RFA elicited significantly higher anti-H3N2 IgG titer than H3N2 vaccine alone at 0.3 µg dose (Fig. 3B). After lethal viral challenges, mice in RFA groups showed significantly slower body weight loss as compared to that in vaccine alone group (Fig. 3C). Mice in vaccine alone group lost a maximum of 20% body weight, while that in RFA groups lost less than 10% body weight (Fig. 3C). The majority of the mice in RFA groups recovered to their original body weights on day 10, while mice in vaccine alone group only recovered to 96% of their original body weights at the end of the study (day 14) (Fig. 3C). All mice in RFA groups and 80% mice in vaccine alone group survived the challenge, while all mice in NI group succumbed to the challenge (Fig. 3D).

**Figure 4:**
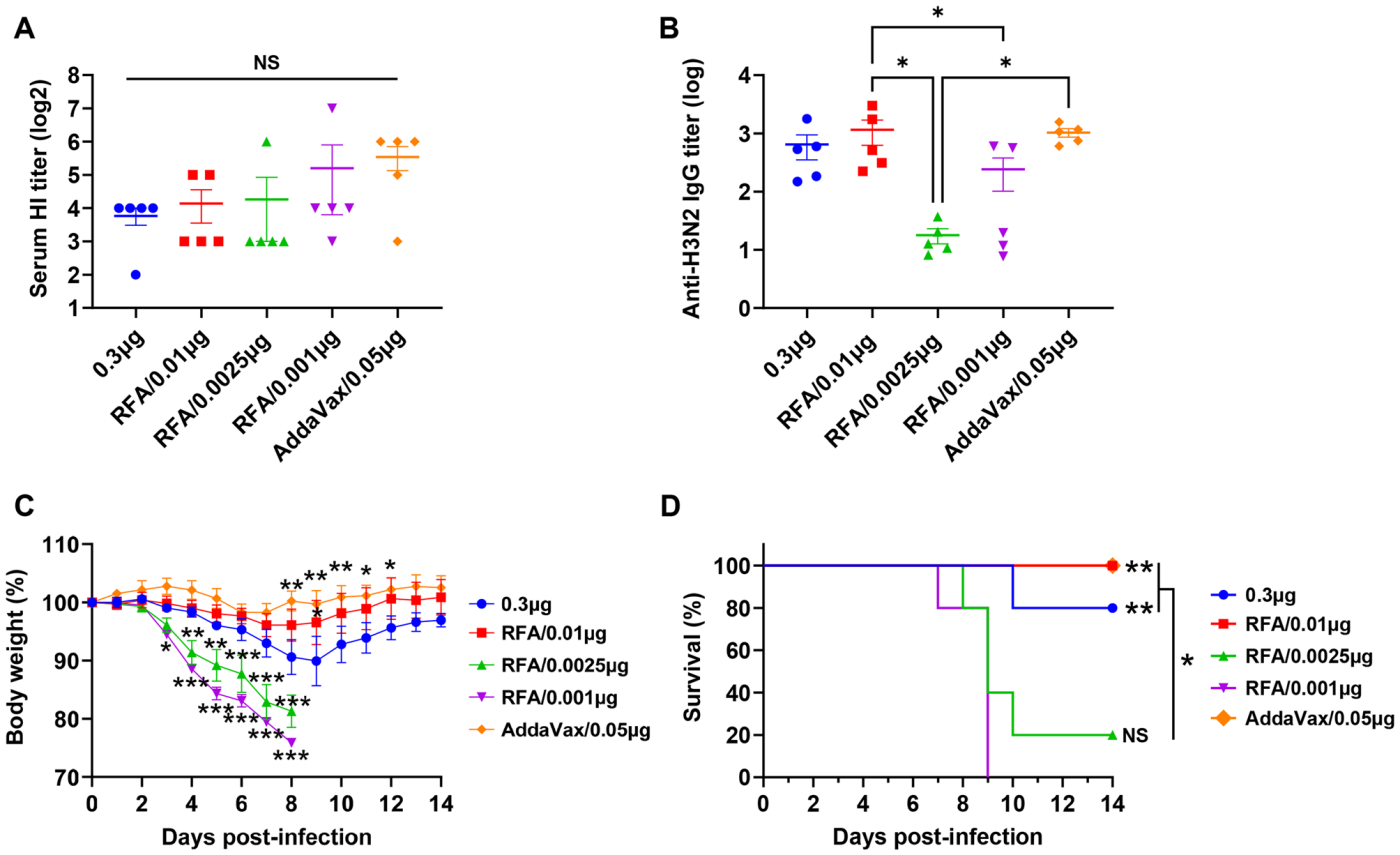
Dose-sparing effects of RFA in vaccine dose range (0.01–0.001 µg). BALB/c mice were intradermally injected with 0.3 µg H3N2 vaccine or subjected to RF treatment followed by ID injection of H3N2 vaccine of further reducing doses (0.01–0.001 µg). Serum HI (**A**) and IgG titers (**B**) were measured three weeks later. Mice were challenged with 50 × LD50 of H3N2 viruses four weeks after immunization. Body weight change (**C**) and survival (**D**) were monitored daily for 14 days. n = 5. One-way ANOVA with Dunn’s multiple comparison test was used to compare differences among groups in (**A**) and (**B**). Two-way ANOVA with Dunnett’s multiple comparison test was used to compare differences between vaccine alone and other groups in (**C**). Log-rank test with Bonferroni correction was used to compare differences of survival between RFA/0.001µg and other groups except otherwise specified in (**D**). *p < 0.05; **p < 0.01; ***p < 0.001. *NS* not significant.

In the second experiment, H3N2 vaccine doses were further reduced to explore the least vaccine dose in the presence of RFA to elicit similar protection to H3N2 vaccine alone at 0.3 µg dose. IM immunization of H3N2 vaccine at 0.05 µg dose in the presence of AddaVax was included for comparison. As shown in Fig. 4A, serum HI titer showed no significant difference among groups. Serum anti-H3N2 IgG titer showed a slightly different trend. There was still no significant difference in serum anti-H3N2 IgG titer between vaccine alone and other groups (Fig. 4B). However, serum anti-H3N2 IgG titer in RFA/0.01 µg group was significantly higher than that in RFA/0.0025 µg and RFA/0.001 µg groups (Fig. 4B). Serum anti-H3N2 IgG titer in AddaVax/0.05 µg was also significantly higher than that in RFA/0.0025 µg group (Fig. 4B). Following lethal viral challenges, mice in RFA/0.01 µg group showed a slower rate of body weight loss than that in vaccine alone group (Fig. 4C), hinting better protection elicited by 0.01 µg vaccine in the presence of RFA. In contrast, mice in RFA/0.0025 µg and RFA/0.001 µg groups showed more rapid body weight loss than that in vaccine alone group (Fig. 4C), hinting inferior protection induced by 0.0025 and 0.001 µg vaccine in the presence of RFA. Similar protection was observed between RFA/0.01 µg and AddaVax/0.05 µg groups, while better protection was observed in AddaVax/0.05 µg group than 0.3 µg vaccine alone group (Fig. 4C). Consistent with body weight data, all mice survived in RFA/0.01 µg and AddaVax/0.05 µg groups and 80% mice survived in vaccine alone group, while only 20% mice survived in RFA/0.0025 µg and all mice died in RFA/0.001 µg group (Fig. 4D). This study indicated 0.01 µg or 10 ng H3N2 vaccine (HA equivalent) could elicit significant protection in the presence of RFA, hinting potent dose-sparing effects of RFA on H3N2 vaccination.

### Crucial roles of HSP70 and MyD88 in dose-sparing effects of RFA

**Figure 5:**
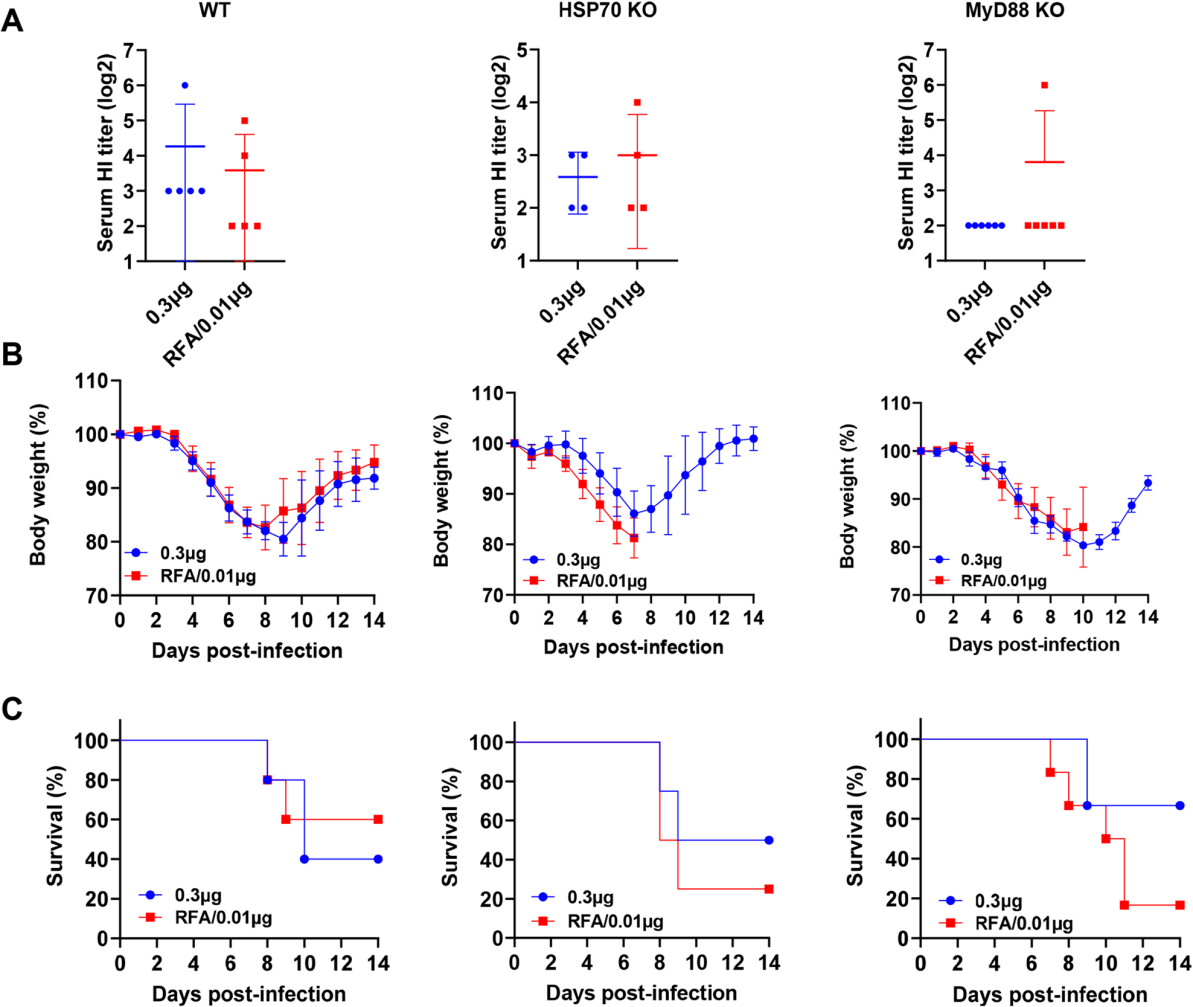
Crucial roles of HSP70 and MyD88 in dose-sparing effects of RFA. WT (C57BL/6), HSP70 KO, and MyD88 KO mice were intradermally injected with 0.3 µg H3N2 vaccine or subjected to RF treatment followed by ID injection of H3N2 vaccine at 0.01 µg dose. Serum HI titers were measured three weeks later (**A**). Mice were challenged with 50 × LD50 of H3N2 viruses four weeks after immunization. Body weight change (**B**) and survival (**C**) were monitored daily for 14 days. n = 5 for WT, n = 4 for HSP70 KO, n = 6 for MyD88 KO. Two-tailed student’s t-test was used to compare differences in (**A**). Two-way ANOVA with Dunnett’s multiple comparison test was used to compare differences between groups at different time points in (**B**). Log-rank test with Bonferroni correction was used to compare differences of survival between groups in (**C**). No significant difference was found in all comparisons in A-C.

Our previous studies found RFA could induce HSP70 release and MyD88 played a crucial role in RFA effects to boost pdm09 vaccination^20^. It remained to be explored whether the above observed potent dose-sparing effects of RFA also depended on HSP70 or MyD88. To explore this, wild type (WT), HSP70 KO, and MyD88 KO mice were intradermally immunized with 0.3 µg H3N2 vaccine or 0.01 µg H3N2 vaccine in the presence of RFA. Serum HI titer was measured 3 weeks later. As shown in Fig. 5A, 0.01 µg vaccine in the presence of RFA elicited similar levels of HI titers to 0.3 µg vaccine alone in WT, HSP70 KO, and MyD88 KO mice. After lethal viral challenges, a similar rate of body weight loss was observed between the two groups in WT, HSP70 KO, or MyD88 KO mice (Fig. 5B). Yet, we observed markedly reduced survival in RFA group in HSP70 KO and MyD88 KO mice (Fig. 5C). In more detail, 2 out of 5 mice survived in vaccine alone group and 3 out of 5 mice survived in RFA group in WT mice (Table 1). Two out of 4 mice survived in vaccine alone group and only one of 4 mice survived in RFA group in HSP70 KO mice (Table 1). In MyD88 KO mice, 4 out of 6 mice survived in vaccine alone group, while only one of 6 mice survived in RFA group (Table 1). Markedly reduced survival of mice in RFA group in HSP70 KO (25%) and MyD88 KO mice (17%) as compared to WT mice (60%) hinted importance of HSP70 and MyD88 in dose-sparing effects of RFA. To be noted, slightly increased survival was observed in vaccine alone groups in HSP70 KO (50%) and MyD88 KO mice (67%) when compared to WT mice (40%) though such a difference was not statistically significant.

**Table 1:** Summary of survival in WT and KO mice.

	WT	HSP70 KO	MyD88 KO
0.3 µg	2/5 (40%)	2/4 (50%)	4/6 (67%)
RFA/0.01 µg	3/5 (60%)	1/4 (25%)	1/6 (17%)

### Cross-protective immunity induced by RFA

**Figure 6:**
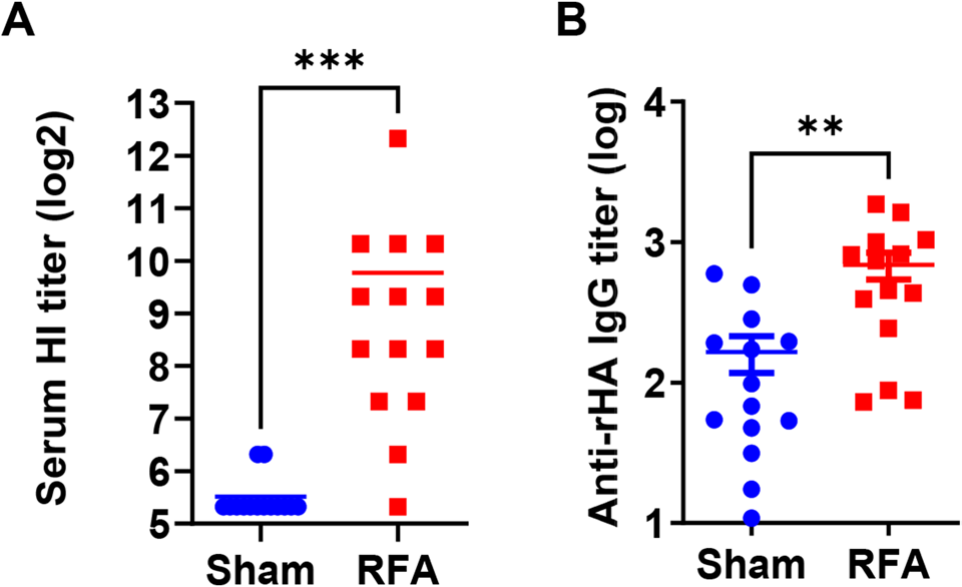
RFA enhances pdm09 vaccine-induced antibody responses. BALB/c mice were subjected to RF or sham treatment followed by ID delivery of 0.3 µg pdm09 vaccine into RF or sham-treated skin. Immunization was repeated 3 weeks later. Serum HI titer (**A**) and anti-rHA IgG titer (**B**) were measured 3 weeks after boost. Two-tailed student’s t-test was used to compare differences between groups. n = 14. **p < 0.01; ***p < 0.001.

Seasonal influenza vaccines usually lack the ability to induce cross-protective immunity. Incorporation of vaccine adjuvants may broaden vaccine-induced immune responses. Next, we explored whether influenza vaccination in the presence of RFA could induce cross-protective immunity. Mice were subjected to RF or sham treatment followed by ID injection of 0.3 µg pdm09 vaccine into RF or sham-treated skin. Immunization was repeated 3 weeks later. As shown in Fig. 6A, ID immunization in the presence of RFA increased serum HI titer by ~ 19 folds as compared to ID immunization alone. RFA also significantly increased serum anti-rHA IgG titer by over 4 folds (Fig. 6B). We also measured HI titer against heterologous PR8 and heterosubtypic H3N2 viruses and found only 1–2 serum samples in RFA group had detectable HI titers, which showed no significant difference from that in sham group (data not shown).

**Figure 7:**
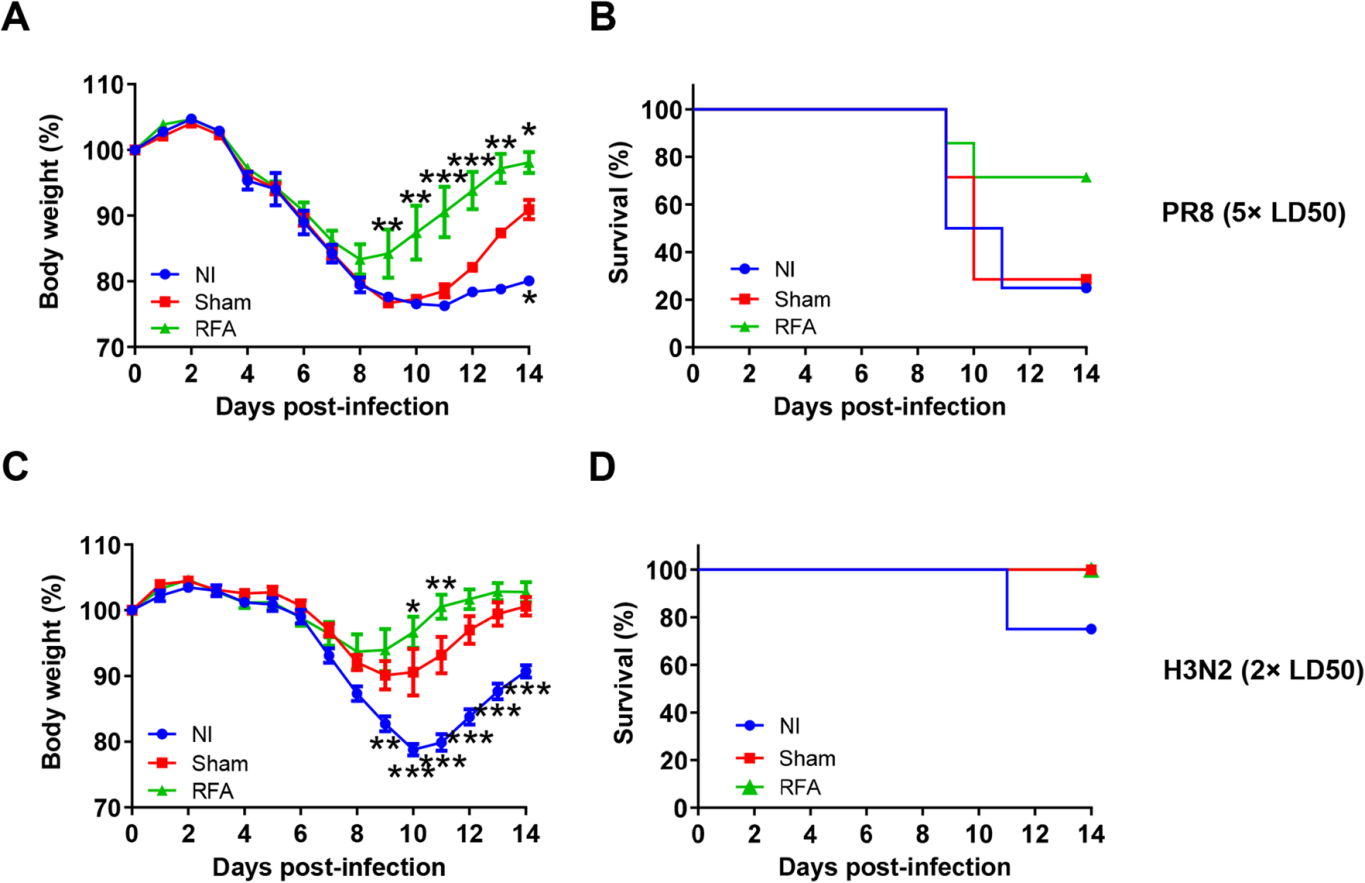
Induction of cross-protection by RFA. (**A**,**B**) BALB/c mice were challenged with 5 × LD50 of PR8 viruses. Body weight (**A**) and survival (**B**) were monitored daily for 14 days. (**C**,**D**) BALB/c mice were challenged with 2 × LD50 of H3N2 viruses. Body weight change (**C**) and survival (**D**) were monitored daily for 14 days. n = 4 for NI; n = 7 for sham and RFA. Two-way ANOVA with Tukey’s post-test was used to compare body weight differences between sham and other groups at different time points in (**A**) and (**C**). *p < 0.05; **p < 0.01; ***p < 0.001.

Mice were then randomly divided into two groups and intranasally challenged with 5 × LD50 of heterologous PR8 viruses or 2 × LD50 of heterosubtypic H3N2 viruses. As shown in Fig. 7A, mice in RFA group showed significantly less body weight loss from day 9 after PR8 viral challenge than mice in sham group. Mice in sham group showed significantly less body weight loss only on day 14 when compared to mice in NI group (Fig. 7A). Consistently, 5 out of 7 mice in RFA group survived PR8 viral challenge, while 2 in 7 mice in sham group and one in 4 mice in NI group survived PR8 viral challenge (Fig. 7B). This study indicated pdm09 vaccine in the presence of RFA induced cross-protective immunity against heterologous PR8 viruses. Following heterosubtypic H3N2 viral challenges, mice in sham group showed reduced body weight loss and mice in RFA group showed more significantly reduced body weight loss as compared to mice in NI group (Fig. 7C). Three out of 4 mice survived in NI group and all mice survived in sham and RFA groups (Fig. 7D). This result indicated pdm09 vaccine in the presence of RFA also elicited cross-protective immunity against heterosubtypic H3N2 viruses.

## Discussion

This study found RFA had potent adjuvant effects to boost seasonal influenza vaccination in murine models. Our prior studies found RFA could significantly boost pdm09, H5N1, and recombinant NP/M1 vaccination. Considering seasonal influenza vaccines post 2009 H1N1 influenza pandemic contain pdm09 or pdm09-like viruses, RFA is expected to similarly enhance seasonal influenza vaccine efficacy against H1N1 viral strains. However, it remained to be explored whether RFA could similarly enhance seasonal influenza vaccine efficacy against H3N2 and type B viral strains. This study showed RFA could enhance seasonal influenza vaccine efficacy against all vaccine viral strains (H1N1, H3N2, type B) though to different extents. RFA induced a similar fold increase of serum HI titer against H1N1 viral strain in this study as compared to our prior study (3.6 vs. 3.44)^20^. Interestingly, RFA more significantly enhanced serum HI titer against H3N2 strain (6.4-fold increase) and less significantly against type B virus (1.75-fold increase). Similar findings were also found when MF59 adjuvant was explored to boost seasonal influenza vaccination. MF59 adjuvant most significantly enhanced HI titer against H3N2 strain and least significantly against type B strain in young children and old adults^28,29^. The underlying reason of the different adjuvant effects on diverse viral strains remained to be explored.

One question in physical adjuvant development is their relative potency to chemical adjuvants. This study compared RFA and MF59-like AddaVax adjuvant to boost H3N2 vaccination and results showed comparable HI titer and similar protection between RFA/ID and AddaVax/IM groups. This result recapitulated our prior finding that ID immunization of 0.3 µg pdm09 vaccine in the presence of RFA elicit similar HI titer and protection to IM immunization of the same dose of pdm09 vaccine in the presence of AddaVax adjuvant^20^. AddaVax was intramuscularly delivered in these studies due to its risk to induce significant local reactions following ID delivery.

Our study discovered robust dose-sparing effects of RFA on monovalent H3N2 vaccination. Ten nanograms of H3N2 vaccine in the presence of RFA elicited better immune responses and protection than 0.3 µg H3N2 vaccine alone. Reduction of vaccine dose to 2.5 ng with RFA elicited weaker immune responses and protection than 0.3 µg H3N2 vaccine alone. This indicates the least H3N2 vaccine dose that RFA is effective to boost is between 2.5 and 10 ng. IM immunization of H3N2 vaccine at 0.05 µg dose in the presence of AddaVax elicited similar immune responses and protection to ID immunization of H3N2 vaccine at 10 ng dose in the presence of RFA. Our prior study found RFA could effectively enhance pdm09 vaccination at 0.06 µg dose, while AddaVax was ineffective at this low dose^20^. These findings indicated RFA had potent dose-sparing effects, superior or at least similar to AddaVax adjuvant. A literature search found rarely nanogram doses of vaccines elicited potent immune responses and protection. One study prepared poly(lactic-co-glycolic acid) (PLGA) nanoparticles coated with PEGylated phospholipid bilayers with MPL further incorporated in the lipid bilayer for conjugation of vaccine antigens^30^. Using this sophisticated system, 2.5 ng ovalbumin was found to induce potent antibody responses in murine models, while at such a low antigen dose Alum and MPL failed to elicit potent immune responses^30^. In another study, AS03B but not AS03A could significantly enhance 3 ng influenza A/Uruguay/716/2007 H3N2 vaccination after one immunization in murine models^31^. Three ng remained the lowest antigen dose that could elicit potent immune responses in the presence of AS03 adjuvant in this study^31^. The ability of RFA to effectively enhance nanogram doses of H3N2 vaccine-induced immune responses and protection hinted its highly potent dose-sparing effects.

Our current study found HSP70 and MyD88 played a crucial role in dose-sparing effects of RFA. Although no significant difference in HI titer, body weight loss, or survival was found between RFA/0.01 µg and 0.3 µg vaccine groups in WT, HSP70 KO, or MyD88 KO mice, significantly reduced survival in RFA/0.01 µg group in HSP70 KO and MyD88 KO mice hinted their crucial role in dose-sparing effects of RFA. It remained to be explored whether HSP70 also played a crucial role in immune-enhancing effects of RFA, which is highly likely considering HSP70 can be released extracellularly with immune-potentiating functions^32,33^. Our prior study and this study found crucial roles of MyD88 in immune-enhancing and dose-sparing effects of RFA^20^. It remained to be explored how RFA stimulated MyD88 pathway due toits physical nature. One possible explanation is RFA stimulates release of endogenous danger signals to indirectly activate MyD88 pathway. It remained to be explored whether HSP70 served as the endogenous danger signal that activated MyD88 pathway.

RFA was further found to assist pdm09 vaccine to induce cross-protective immunity. As compared to pdm09 vaccine alone, pdm09 vaccine in the presence of RFA conferred better protection against heterologous PR8 viral challenges and slightly better protection against heterosubtypic H3N2 viral challenges. These results indicated RFA elicited more potent heterologous protection than heterosubtypic protection in murine models, which could be explained by higher HA homology between heterologous H1N1 strains than between heterosubtypic strains (H1N1 and H3N2). Induction of cross-protective immunity is highly desired for influenza vaccination due to the constant mutation of influenza viruses, which requires the current influenza vaccines to be updated annually. Regarding cross-protective mechanisms, it might be due to the induction of cross-reactive cytotoxic T lymphocyte (CTL) responses in the absence of significant HI titers elicited against heterologous or heterosubtypic viral strains (data not shown). In support, our prior studies found RFA could elicit potent CTL responses against OVA, rHA, and NP/M1^18,20^.

Our current study strongly supports further development of RFA to boost influenza vaccination. Prior to RFA, various types of laser were explored to boost ID vaccination. Laser adjuvants emit green or near-infrared light on a small area of the skin to enhance dendritic cell (DC) migration or transportation to draining lymph nodes to enhance ID vaccine-induced immune responses^34–42^. Non-ablative fractional laser (NAFL) was also explored to boost ID vaccination^43,44^. NAFL induces microscopic skin damage and was found to recruit plasmacytoid DCs (pDCs) and induce dsDNA release to mediate its adjuvant effects^43,45^. Prior to laser adjuvants, low-frequency sonophoresis (LFS) was found to activate epidermal Langerhans cells and at the same time breach the barrier function of the skin to facilitate transcutaneous immunization^46^. To our knowledge, this is the first report that a physical adjuvant (RFA) elicits such a potent dose-sparing effect and also induces cross-protective immunity against influenza vaccination. RFA was also highly potent and could significantly enhance ID pdm09 and H3N2 vaccine-induced immune responses and protection to a similar extent to that induced by IM vaccination in the presence of MF59-like AddaVax adjuvant.

## Data Availability

The raw/processed data required to reproduce these findings are available from the corresponding author upon request.
